# A CRISPR interference strategy for gene expression silencing in multiple myeloma cell lines

**DOI:** 10.1186/s13036-023-00347-7

**Published:** 2023-05-04

**Authors:** Josep Escrivá-Fernández, Cristina Cueto-Ureña, Amalia Solana-Orts, Elisa Lledó, Begoña Ballester-Lurbe, Enric Poch

**Affiliations:** 1 Department of Biomedical Sciences, School of Health Sciences, Universidad Cardenal Herrera-CEU, CEU Universities, Alfara del Patriarca, E-46115 Valencia, Spain; 2 Experimental and Clinical Physiopathology Research Group CTS-1039, Department of Health Sciences, School of Health Sciences, University of Jaén, E-23071 Jaén, Spain; 3 Department of Biomedical Sciences. School of Health Sciences, Universidad Cardenal Herrera-CEU, C/ Ramón y Cajal s/n, E-46115 Alfara del Patriarca, Valencia, Spain

**Keywords:** Multiple myeloma, *RND3*/Rnd3, CRISPRi, CRISPR/Cas9, Lentiviral transduction

## Abstract

**Background:**

Multiple myeloma (MM) is the second most common hematologic neoplasm which is characterized by proliferation and infiltration of plasmatic cells in the bone marrow. Currently, MM is considered incurable due to resistance to treatment. The CRISPR/Cas9 system has emerged as a powerful tool for understanding the role of different genetic alterations in the pathogenesis of hematologic malignancies in both cell lines and mouse models. Despite current advances of gene editing tools, the use of CRISPR/Cas9 technology for gene editing of MM have not so far been extended. In this work, we want to repress Rnd3 expression, an atypical Rho GTPase involved in several cellular processes, in MM cell lines using a CRISPR interference strategy.

**Results:**

We have designed different guide RNAs and cloning them into a lentiviral plasmid, which contains all the machinery necessary for developing the CRISPR interference strategy. We co-transfected the HEK 293T cells with this lentiviral plasmid and 3rd generation lentiviral envelope and packaging plasmids to produce lentiviral particles. The lentiviral particles were used to transduce two different multiple myeloma cell lines, RPMI 8226 and JJN3, and downregulate Rnd3 expression. Additionally, the impact of Rnd3 expression absence was analyzed by a transcriptomic analysis consisting of 3’ UTR RNA sequencing. The Rnd3 knock-down cells showed a different transcriptomic profile in comparison to control cells.

**Conclusions:**

We have developed a CRISPR interference strategy to generate stable Rnd3 knockdown MM cell lines by lentiviral transduction. We have evaluated this strategy in two MM cell lines, and we have demonstrated that Rnd3 silencing works both at transcriptional and protein level. Therefore, we propose CRISPR interference strategy as an alternative tool to silence gene expression in MM cell lines. Furthermore, Rnd3 silencing produces changes in the cellular transcriptomic profile.

**Supplementary Information:**

The online version contains supplementary material available at 10.1186/s13036-023-00347-7.

## Background

Multiple myeloma (MM) is a hematologic neoplasm of plasmatic cells that infiltrates the bone marrow and secretes monoclonal immunoglobulins. MM is the second most common hematologic malignancy in the world, and despite the therapeutic advances, the disease is associated with a poor outcome, due to disease relapsing and the development of treatment resistances [1–3].

MM is preceded by a pre-malignant and asymptomatic stage called monoclonal gammopathy of undetermined significance (MGUS) and intermediate stage termed smoldering multiple myeloma (SMM). Then, most patients develop the symptomatic stage known as MM and characterized by the monoclonal protein secretion and the end-organ damage [1, 2]. Finally, MM cells can develop the ability of proliferation outside the bone marrow and produce more aggressive stages such as extramedullary multiple myeloma (EMM) or plasma cell leukemia (PCL) [1, 2].

How the asymptomatic MGUS stage becomes a MM remains unknown, but many factors are involved, being the genetic alterations the most frequent causes of disease progression. Also, cells present in the bone marrow microenvironment play an important role in MM progression. These cells, that include bone marrow stromal cells, endothelial cells, and other hematopoietic cells, produce chemokines and other factors that interact with MM cells favoring their migration to the bone marrow and facilitating their proliferation and survival [4].

Rho GTPases are small GTP-binding proteins with an important role in converting extracellular signals into a large range of cellular responses, including cell adhesion, cell-cycle progression, cell migration, cell morphogenesis, gene expression, and actin cytoskeleton dynamics [5–7]. Therefore, Rho proteins have been widely described as important regulators of tumour cell proliferation, survival, and invasion [8]. Rho proteins also play an important role in the development of hematological neoplasms since they are involved in chemotaxis and motility in lymphoid lines through the ROCK-LIMK pathway [9]. Specifically, RhoU, an atypical GTPase Rho protein, has been recently related to MM progression, as its expression increases in MGUS patients, and it is downregulated as the disease progresses [10]. Rnd3 protein is an atypical GTPase that belongs to Rho family and it has been widely associated to diverse cellular processes such as cell polarity and differentiation, survival, proliferation and migration [11]. Moreover, and based on our and other research groups results, alterations in Rnd3 expression have been related to several physiological disorders, including cancer progression and tumor drug resistance [12, 13]. However, the possible role of Rnd3 in hematological neoplasm remains unknown, so our investigation aims to determine the potential role of Rnd3 in MM. For this reason, a cellular model lacking Rnd3 must be obtained to determine the impact of the absence of Rnd3 in some important processes during MM progression, such as proliferation, adhesion, cell migration and others. Among all the biotechnological tools available, we decided to combine the specificity of the CRISPR/Cas9 system with the use of lentiviral vectors to optimize the success of *RND3* gene knock down.

The CRISPR/Cas9 system has emerged as a powerful tool for understanding the role of different genetic alterations in the pathogenesis of hematologic malignancies, as well as discovering new therapeutic targets for future clinical stages [14, 15]. The CRISPR system is a bacterial adaptive immune system that requires the endonuclease Cas9 from *Streptococcus pyogenes* (or analogous proteins from other species) and a single guide of RNA (sgRNA) [16]. That guide leads the nuclease activity to complementary sequences in the substrate DNA, usually on the coding region [17]. CRISPR/Cas9 is used for genome editing by introducing deletions on the protein coding ORF (open reading frame) using homology repair (HR) or nonhomologous end joining (NHEJ) repair. These deletions can lead to frame shifts that result in a loss of function of the encoded protein [18]. CRISPR/Cas9 is a permanent technique that allows the generation of knockout cell lines by genome editing, but it has variable tolerance for mismatches between its sgRNA and the target DNA sequence, so off-targets effects are common with this technology [19]. For this reason, variants of this technique have been developed as one that contains a catalytically inactivated Cas9, known as a dead Cas9 (dCas9). This dCas9 can be used to activate or repress gene expression when it is associated to a sgRNA directed to specific gene promoter region. If the dCas9 is fused to a transcriptional activator such as VP64, the gene expression is activated, and it is known as CRISPR activation. Also, dCas9 can be fused to a transcriptional repressor, known as Krüppel-associated box (KRAB), that can induce DNA methylation and decrease the accessibility of chromatin at the enhancer and promoter regions and, therefore, represses gene expression at transcriptional level. This technology is called CRISPR interference (CRISPRi) [20, 21] and has demonstrated a high degree of efficiency in gene silencing, without manipulation of the cell’s or organism’s DNA. CRISPRi is frequently used to perform genetic screens in mammalian cells [21] and is also used to cell engineering and regenerative medicine like retinal, muscle, nerve, or bone degeneration among others [20]. Furthermore, CRISPRi can be used to silencing gene expression in specific human cell types, such as neurons or iPSCs [22, 23]. This silencing can be constitutive or inducible, but frequently it is stable when it is achieved by lentiviral transduction.

In this work, we describe and analyze the efficacy of CRISPRi technology when it is used to silencing Rnd3 expression in MM cell lines by lentiviral transduction.

## Results

### sgRNAs cloning into lentiviral plasmid pLV hU6-sgRNA hUbC-dCas9-KRAB-T2a-Puro

To obtain the sequence of the sgRNA an Addgene library (CRISPRi library was a gift from Jonathan Weissman (Addgene #62217)) was consulted [24]. From the sequences available directed to *RND3* promoter, two different sgRNA were selected. In addition, another sgRNA guide to a non-coding region (scrambled) was chosen to be used as negative control. To allow the cloning with the selected sgRNAs, a prior modification must be performed. In the forward and reverse primers, a nucleotide tails, target of Esp3I restriction enzyme must be introduced (Table 1).

**Table 1:** List of primers used to generate the sgRNAs. The necessary tails in the ends for the cloning were highlighted

Name	Sequence (5’→3’)
**sgRND3 forward #3**	**CACCG**GAAACGCGGCGCAGACGAGG
**sgRND3 reverse #3**	**AAAC**CCTCGTCTGCGCCGCGTTTC**C**
**sgRND3 forward #4**	**CACCG**GGGACTTGGGAGGCGCGGTG
**sgRND3 reverse #4**	**AAAC**CACCGCGCCTCCCAAGTCCC**C**
**sgRNA Scramble forward**	**CACCG**GGAGGACGATCGTACTCCAG
**sgRNA Scramble reverse**	**AAAC**CTGGAGTACGATCGTCCTCC**C**

**Fig. 1:**
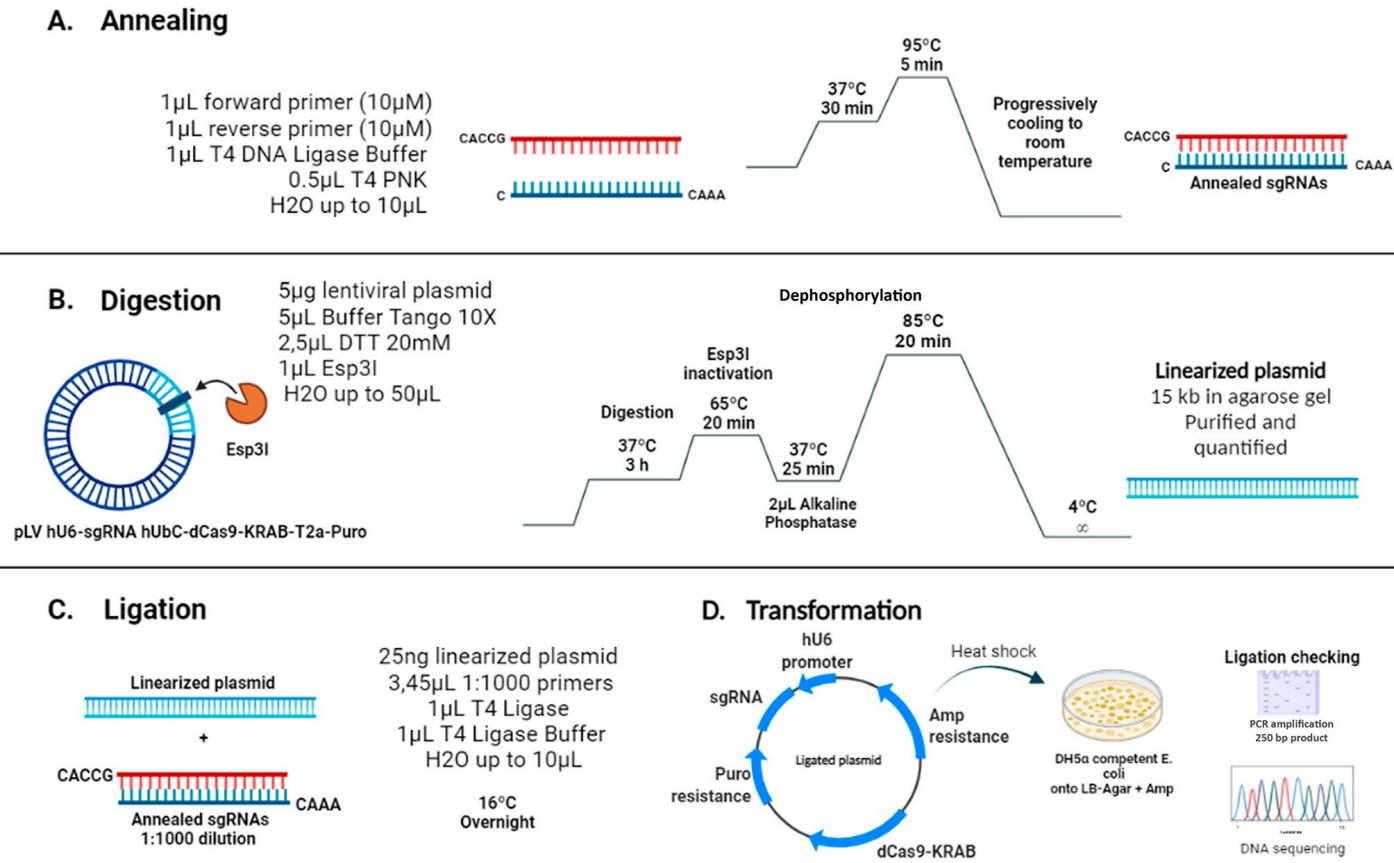
Schematic protocol of sgRNA cloning into lentiviral plasmid. First, the annealing of the primers was required to generate the sgRNAs **(A)**. Then, the lentiviral plasmid was digested with Esp3I enzyme to linearize, purify and quantify **(B)**. The ligation of the sgRNA into the linearized lentiviral plasmid was made at 16ºC overnight in presence of T4 ligase enzyme **(C)**. Finally, the ligated plasmids were transformed in DH5α competent *E. coli* **(D)** and colonies were checked by PCR and sequencing, before DNA amplification.

The annealing of the designed primers was necessary to prepare a double stranded sgRNAs. This process requires the use of forward and reverse oligonucleotides, and their incubation at 95ºC, allowing a progressive cooling. Finally, T4 PNK enzyme was added to phosphorylate the 5’ end for subsequent cloning ligation. The details of the process are described in Fig. 1A. Then, the annealed sgRNAs were diluted 1:1000 and cloned into lentiviral plasmid construction pLV hU6-sgRNA hUbC-dCas9-KRAB-T2a-Puro (this plasmid was a gift from Charles Gersbach (Addgene plasmid # 71236); [25]). 5 µg of plasmid was linearized with Esp3I enzyme (Thermo Fisher) and then it was dephosphorylated with alkaline phosphatase (Promega) (Fig. 1B). The digested plasmid was analyzed using an agarose gel electrophoresis and the 15 kb expected band was purified using NZYGelpure kit (Nzytech), following the manufacturer’s instructions and quantified by Nanodrop Simplinano (GE Healthcare Life Science). The ligation reaction containing both the linearized plasmid and the annealed sgRNA was prepared and incubated overnight as described in Fig. 1C. Finally, the ligation product was transformed into DH5α competent *E. coli* and grown on to LB-Agar plates with ampicillin. To check the ligation procedure, plasmid DNA was obtained using GenElute Plasmid Miniprep kit (Sigma-Aldrich) and two different strategies were carried out: an amplification using a PCR with specific primers and DNA sequencing (Fig. 1D). Once checked, bacteria culture was amplified to obtain enough plasmid DNA using PureLink Plasmid Filter Midiprep kit (Invitrogen) following the manufacturer’s instructions. These plasmids containing the sgRNAs cloned into the lentiviral vector were then used to be transfected in HEK 293T cells.

### Transfection, production and concentration of lentiviral particles

The production of lentiviral particles was carried out by the calcium phosphate transfection method. HEK 293T lentiviral packaging cells were cotransfected with different vectors that included the sgRNA plasmids and 3rd generation lentiviral envelope and packaging plasmids (Table 2). 24 h prior the transfection, HEK 293T cells were plated in 10 cm plates and reached 60–70% of confluence the day of transfection. Two hours before transfection the medium was removed and replaced with 9 mL of fresh medium. To obtain a high virus titer, five 10 cm plates per each sgRNA construction were needed although **the information related to the volumes and quantities hereby is referred to one 10 cm culture plate.**

**Table 2:** List of plasmids used for HEK 293T cells co-transfection

Name	Function	Description
**pVSV-G Retroviral vector (pVSV-G)**	Envelope	Expresses the G glycoprotein of the vesicular stomatitis virus, necessary for viral entry
**pMDLg-pRRE (pRRE)**	Packaging	Includes gag, coding for the virion main structural proteins; pol, responsible for the retrovirus-specific enzymes; and RRE, a binding site for the Rev protein
**pRSV-REV (pREV)**	Packaging	Expresses Rev protein, which facilitates export of the RNA from the nucleus
**pLV hU6-sgRND3 #3 hUbC-dCas9-KRAB-T2a-Puro (sgRND3 #3)**	Silencing *RND3* expression	Expresses all machinery for CRISPRi technique: sgRNA, dCas9 and KRAB
**pLV hU6-sgRND3 #4 hUbC-dCas9-KRAB-T2a-Puro (sgRND3 #4)**	Silencing *RND3* expression	
**pLV hU6-sgRNA Scramble hUbC-dCas9-KRAB-T2a-Puro (Scramble)**	Transduction control	

Every plasmid containing the different *RND3* sgRNA was co-transfected with 3rd generation lentiviral plasmids (3 µg pENV, 5 µg pRRE and 2,5 µg pREV) in a 15 ml tube, together with 15 µg sgRNA plasmid, in a 150 µL final volume placed in a 15ml tube. Then, 300 µL of TE 0.1X and 50 µL of CaCl_2_ were added and the DNA mix was incubated 5 min at room temperature. Finally, 500 µL of HBS 2X was drop by drop added to the mixture while vortexed and rapidly transferred to the HEK 293T cells plate. After 14–16 h the transfection medium was removed, 6 ml of fresh medium was added, and cells were kept for 48 h more. Then, to concentrate the lentiviral particles, the medium from the five plates was recollected and centrifuged for 10 min at 2000 g to discard remaining cells and the supernatant was filtered with 0.45 μm filter. To obtain high virus titer, the filtered supernatant was ultracentrifuged at 125,000 g for 150 min at 4ºC using the Optima L-100 XP ultracentrifuge (SW32 rotor, Beckman Coulter) and the pellet was resuspended in 1 ml of PBS for 30–60 min on ice, with occasional mixing. The lentiviral particles were used to freshly transduce the MM cells or they were frozen at -80ºC to further infections.

### Transduction of MM cells and obtention of Rnd3 knock-down (KD) stable cell lines

RPMI 8226 and JJN3 cells were transduced with concentrated lentiviral particles in 6-well plates. 100 µL of lentiviral particles and 1 µL of polybrene solution (10 mg/mL, Sigma-Aldrich) were added to 1 × 10^6^ MM cells/well in 1ml final volume and cells were kept at 37ºC and 5% CO_2_ for 48 h. Then, the transduction medium was removed, and MM cells were maintained in fresh medium. The selection of transduced cells was made by addition of puromycin (2 µg/mL, Sigma-Aldrich) to the culture medium for 24 h. Finally, puromycin resistant cells were amplified and considered as a stable cell line after sequential passages and used to perform all the experiments. To perform the qPCR and western blot experiments, cells were grown to confluence and RNA or protein extracts were collected two weeks post-transduction.

At the end of this process, we obtained two Rnd3-deficient lines (sgRND3 #3 and #4) for each cell type, together with a scramble line in the case of RPMI cells. In the case of JJN3 cells, it was not possible to obtain a scramble line, so the results shown below were compared with wildtype cells.

**Fig. 2:**
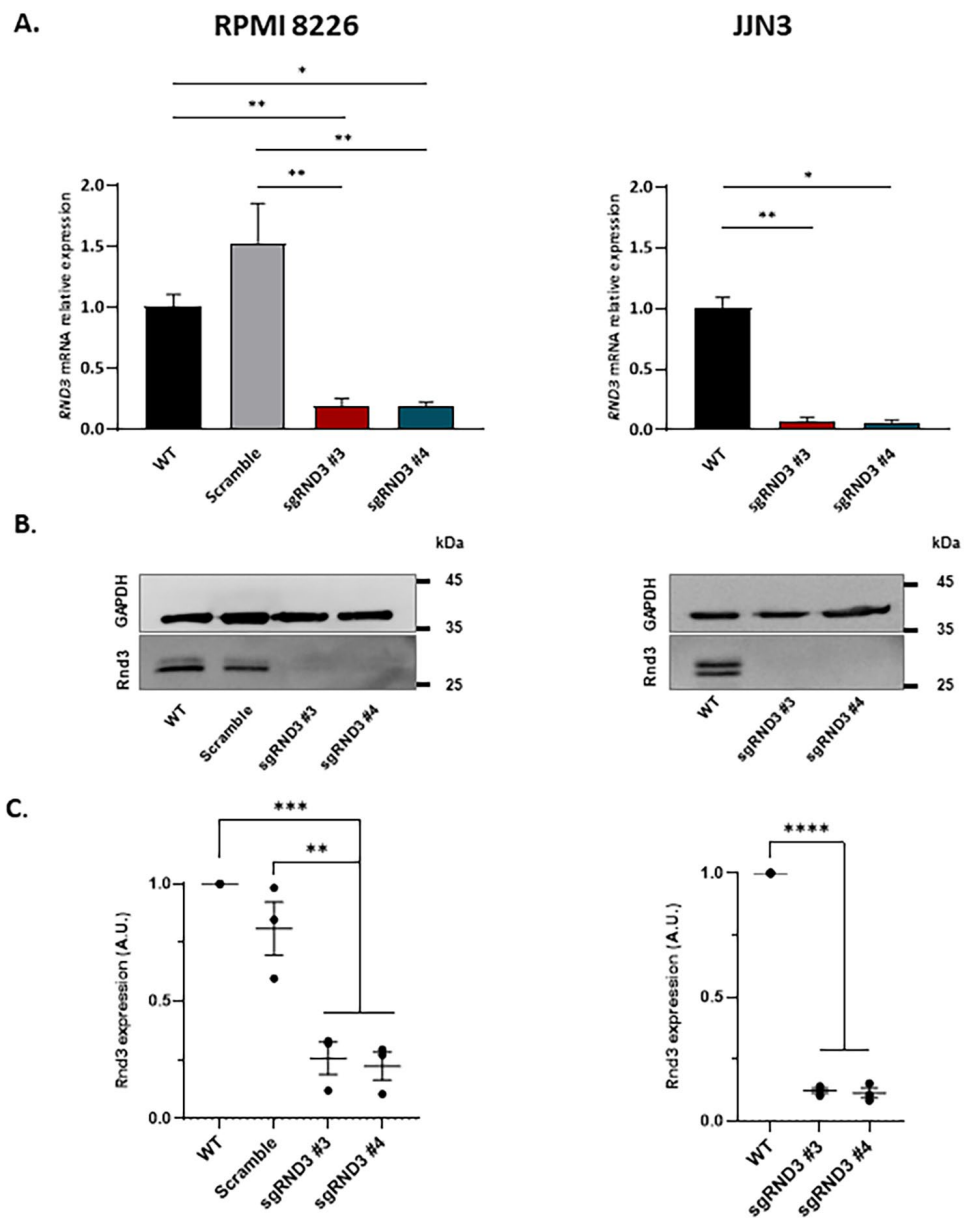
Rnd3 expression was reduced in RPMI 8226 and JJN3 transduced cells. RPMI 8226 and JJN3 *RND3* mRNA expression levels were analyzed by qPCR and calculated as fold change using the 2^−ΔΔCt^ method **(A)**. Representative western blot images of Rnd3 protein expression in RPMI 8226 and JJN3 cells **(B)**. Bands were quantified using ImageJ software and the densitometric values were normalized to GAPDH and plotted in **(C)**. Mean values from 3 independent experiments are plotted and one-way ANOVA and Tuckey’s post-hoc test show differences between groups: *p < 0.05; **p < 0.01, ***p < 0.001, ****p < 0.0001

CRISPRi technology allows an efficient gene silencing at transcriptional level, leading the dCas9-KRAB to the gene promoter and inhibiting its expression as described above. As shown in Fig. 2A, RPMI cells transduced with *RND3* sgRNA lentiviral constructs dramatically decrease *RND3* RNA levels compared to either scramble and wildtype cells (1, 1.5 and 0.2-fold change in wildtype, scramble and both *RND3* sgRNA, respectively). Same results were obtained with JJN3 *RND3* KD cells, showing a 0.06-fold change reduction compared to wildtype cells.

According to the decrease of RNA levels, *RND3* knockdown cells also show a reduction of Rnd3 protein expression (Fig. 2B). Densitometric quantification of immunoblots reveals that RPMI cells lacking Rnd3 decrease up to 75–78% the levels of protein, compared to wildtype cells. In JJN3 cells, gene silencing seems to be more effective, observing a reduction of 88–89% in protein levels compared to wildtype cells (Fig. 2C). After the verification of gene silencing at transcriptional and translational levels, each cell line was frozen for next experiments.

**Fig. 3:**
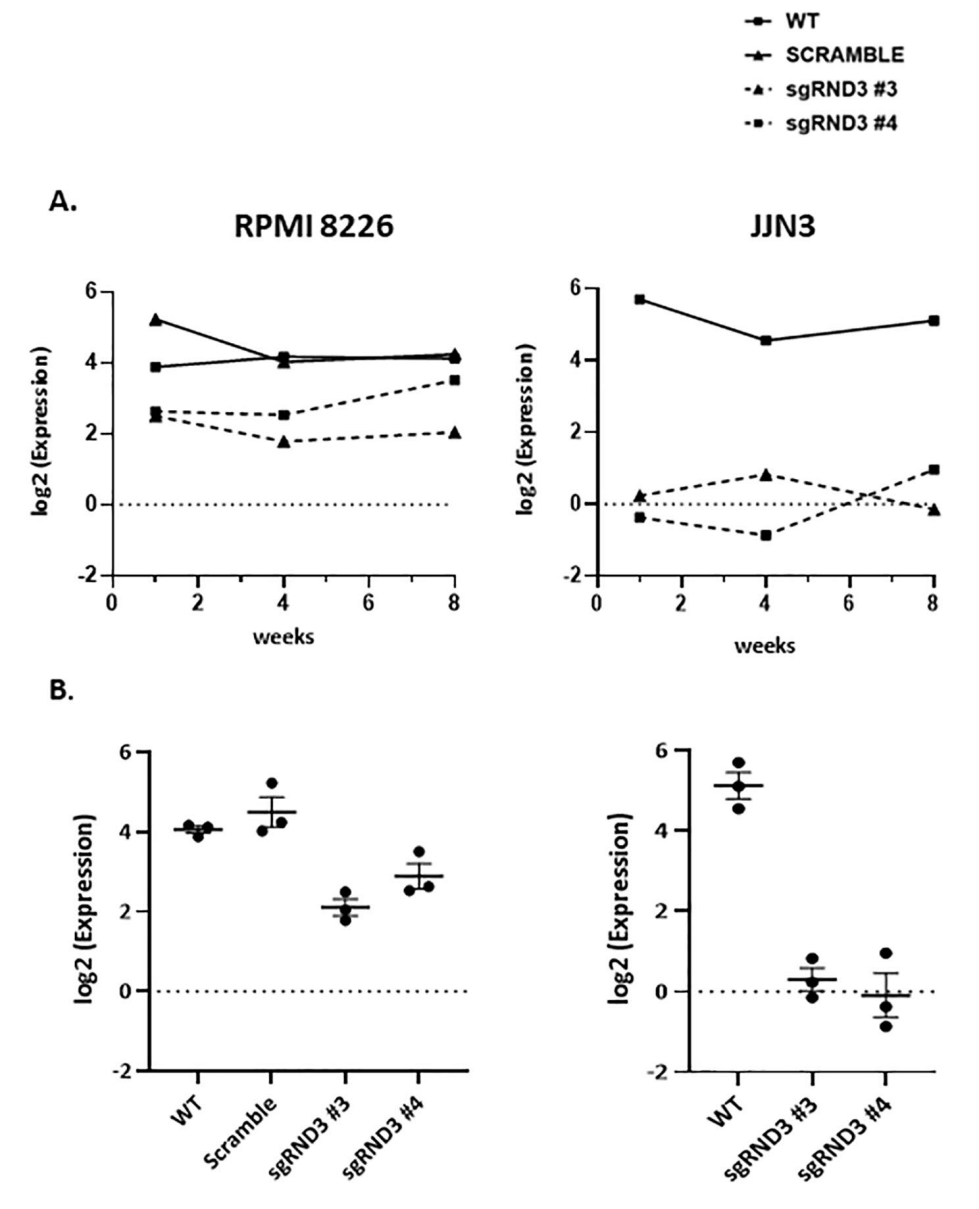
Long-term maintenance of RND3 silencing is observed in RPMI 8226 and JJN3 transduced cells. RNA extracts were obtained from RPMI 8226 and JJN3 cells at different times (1 week, 4 weeks and 8 weeks post-thawing) and RND3 expression was analyzed by RNA sequencing. **(A)** Rnd3 time course expression in either RPMI and JJN3 cells. Values plotted represent single RNA levels for every cell line at the indicated times. **(B)** Mean of the values shown in A) grouped for each time and cell line were also plotted to confirm the consistency of the gene silencing along the time. RPMI 8226 knock-down cells show two-fold change expression decrease (p = 0,0009) while JJN3 knock-down show five-fold change expression decrease (p = 0,0002), compared to WT or scramble cells

To confirm long-term maintenance of gene silencing, transduced cell lines were thawed and collected at 1, 4 and 8 weeks after. Then, *RND3* expression was quantified using a transcriptomic analysis consisting of 3’ UTR RNA sequencing, as described in Methods. As shown in Fig. 3, both cell lines transduced with *RND3* sgRNA lentiviral constructs show a reduction in *RND3* expression compared to WT or Scramble cells over time (Fig. 3A). When the data corresponding to each condition were pooled and plotted together, RPMI 8226 cells show two-fold change decreasing expression (p = 0,0009), while JJN3 show five-fold change decreasing between control and transduced cells (p = 0,0002). Taken together, these results confirm that a significant silencing was achieved using this protocol.

### Rnd3 KD cells shown changes in transcriptomic profile

**Fig. 4:**
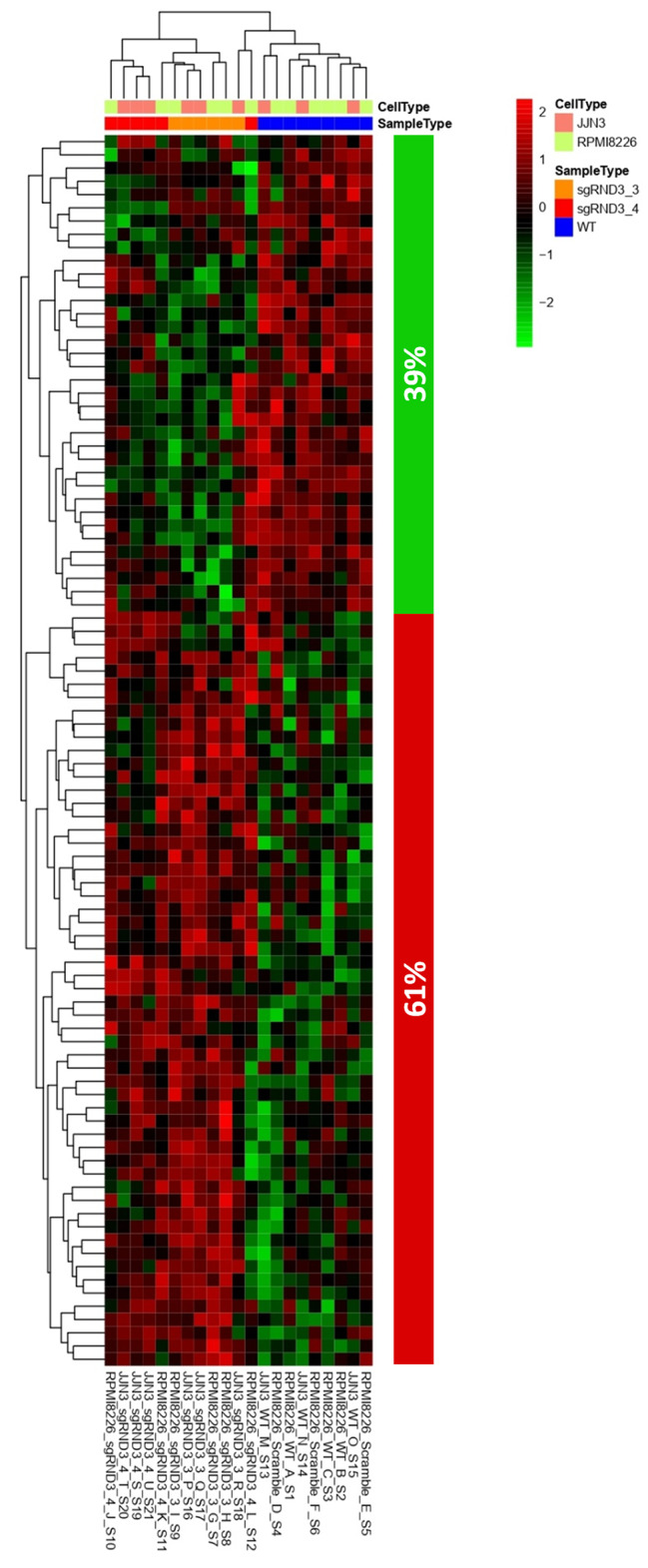
Rnd3 loss-of-function results in gene expression repression signature. Heatmap showing the RNA-seq data of 93 differentially expressed genes upon Rnd3 KD in RPMI 8226 and JJN3 cell lines. Columns represent individual samples; rows correspond to the genes. Heat map represents the z-scores of the expression value (RPKM) characterized by RNA-seq. The column on the right represents the proportion of the genes that were repressed (green) or upregulated (red) in RND3 knockdown cells as compared with scramble or control samples

The results obtained comparing the transcriptomic profile of control (wildtype and/or scramble) versus Rnd3 KD cells showed 93 genes differentially and consistently expressed in both RPMI 8226 and JJN3 cell lines (Fig. 4). Among these genes, specifically 39% were down-regulated and 61% were up-regulated in Rnd3 KD cells. The Gene Ontology (GO) database was used to carry out an enrichment study according to the categories based on the biological processes associated with these genes. With this criterion, the genes that presented significant differences between both groups were selected. Finally, genes associated with biological processes without relevance in MM were discarded. Based on the information obtained, we established a list of 19 differentially expressed genes that were grouped into 6 functional categories: calcium ion transport and mobilization, proinflammatory cytokine production, cell migration and motility, cell-cell interactions, angiogenesis, and cAMP cell signaling. All these functional categories correspond to cellular processes that have been described as relevant in the pathophysiology of MM. These results confirm that Rnd3 silencing produces downstream gene expression changes in MM cells and validates the methodology here proposed. However, further experiments should be addressed to confirm the potential role of Rnd3 in MM etiology, such as the analysis of Rnd3 expression in MM patients.

## Discussion

Gene editing and transcriptomic regulation approaches have become a powerful tool for basic and clinical settings in cellular therapies for lymphoid malignancies. Considering different techniques for gene silencing, RNAis have been used in multiple studies with mammalian cells. Specifically, this strategy has been used by many authors to downregulate the expression of specific genes in MM cell lines [10, 26–30]. RNAi technology acts at translational level because it facilitates the mRNA degradation, so it produces a transient gene silencing. Despite of the advantages that this technique can provide it is described that could generate off-targets [31] that reduces the specificity of the gene repression. Therefore, other strategies to repress gene expression in MM cell lines have been tested such as CRISPR/Cas9.

The CRISPR/Cas9 system has been emerged as a powerful gene editing or transcriptomic regulation tool for basic and clinical settings in cellular therapies for lymphoid malignancies [15]. CRISPR/Cas9 technique allows gene editing using the Cas9 endonuclease and sgRNAs to introduce deletions on the ORF. Nowadays, there are many studies that use CRISPR/Cas9 technology to repress gene expression in human cell lines and provides stable knockout cells for the study of pathologies progression [32]. Despite the real advantages of the CRISPR/Cas9 genome editing, the production of off-target effects is common [19]. Consequently, other strategies that could reduce these off-target effects have been proposed such as high-fidelity Cas9 for genome editing or dCas9 for gene silencing. CRISPR interference (CRISPRi) technology consists in using a sgRNA to direct a dCas9 fused to KRAB repressor to the gene promoter and downregulate gene expression at transcriptional level [21]. Recently, many studies have been developed to inactivate the gene expression in human cells, like neurons or iPSCs [22, 23].

Although the recent advances of gene editing and/or silencing tools, the use of CRISPR/Cas9 technology for gene editing in MM has not so far been extended. A recent study shows the generation of FAM46C knockout MM cell lines using CRISPR/Cas9 [33]. In this work, we propose an extensive protocol for the using of CRISPRi technology to downregulate Rnd3 expression in RPMI 8226 and JJN3 MM cell lines, by using lentiviral vectors and specific *RND3* sgRNA. Our results demonstrate that transduced cells present a significant reduction of Rnd3 expression both at mRNA and protein level, thus allowing to generate stable knock-down cells.

Based on the results here shown, we propose the use of this protocol to specifically knock-down a target gene in MM cells that, based on our experience, refer low transduction efficiency.

## Conclusions

We have developed and validated an effective CRISPRi strategy using lentiviral transduction for gene silencing in multiple myeloma cell lines. This strategy combines the design of the sgRNAs, their cloning into the lentiviral vector with CRISPRi machinery, the transfection of HEK 293T cells to produce the lentiviral particles and the transduction of MM cell lines with these particles for gene silencing. Also, we have demonstrated that this silencing is efficient and works both at mRNA and protein level, so this CRISPRi strategy allows the generation of stable knockdown cell lines. Finally, we have performed a RNAseq analysis indicating that Rnd3 silencing produces changes in the cellular transcriptomic profile of either RPMI 8226 and JJN3 cells, thus demonstrating the potential value of our experimental approach.

## Methods

### Cell cultures

MM cells (RPMI 8226 and JJN3) were used in this work to silence the expression of Rnd3, and human embryonic kidney 293T cells (HEK 293T) to produce lentiviral particles. RPMI 1640 (Gibco, Thermo Fisher), IMDM (Gibco, Thermo Fisher Each) and DMEM (Gibco, Thermo Fisher) were used for RPMI 8226, JJN3 and HEK 293T cells respectively. All media were supplemented with 10% Fetal Bovine Serum (FBS, Gibco, Thermo Fisher) and 1% of penicillin/streptomycin (Gibco, Thermo Fisher), and cells were maintained at 37ºC and 5% CO_2_.

### Total RNA extraction, cDNA synthesis and qPCR

For total RNA extraction, 5 × 10^5^ RPMI 8226 or JJN3 cells were collected and washed with PBS. RNA extraction was performed using NZY total RNA Isolation kit (Nzytech) following the manufacturer’s instructions. Total RNA samples were quantified by optical density at 260/280 nm with Nanodrop Simplinano (GE Healthcare Life Science), and 1 µg was used for cDNA synthesis using the NZY First-Strand cDNA Synthesis kit (Nzytech). Finally, qPCR was made using the cDNA samples with the corresponding primers **(**Table 3**)** and the commercial mix NZYSpeedy qPCR Green Master Mix (2x), ROX (Nzytech), which include a green intercalating dye, dNTPs, stabilizers, and enhancers. Human Large Ribosomal Protein (RPLP0) was used as a housekeeping gene. The qPCR was performed using an AriaMx machine (Agilent Technologies) and Agilent Aria 1.71 software (Agilent Technologies) for gene expression analysis. RND3 mRNA expression levels were analyzed and calculated as fold change using the 2^−ΔΔCt^ method.

**Table 3:** List of primers for the qPCR

Gene	Sequence (5’-->3’)	Product length
***RND3*** **forward**	GCAGACGCCAGTGTCCTAT	192 pb
***RND3*** **reverse**	ATCCGCTTTGTGGCTCTCTG	
***RPLP0*** **forward**	ACAACCCAGCTCTGGAGAAA	240 pb
***RPLP0*** **reverse**	TGCCCCTGGAGATTTTAGTG	

### Protein extraction and western blot

For protein extraction, 1 × 10^6^ cells were collected by centrifugation and washed with PBS. Then, samples were lysated with RIPA buffer (150 mM NaCl, 1% NP40, 0.5% sodium deoxycolate, 0.1% SDS, 50 mM Tris pH 8), protease inhibitors (Complete Tablets Mini EDTA-free, Roche) and phosphatase inhibitors (10 mM NaF, 1 mM Na_3_VO_4_, 1µM DTT). Finally, samples were quantified using Bradford assay (Bio-Rad) and the absorbance at 595 nm was quantified by using a Victor X3 spectrophotometer (Perkin Elmer).

Protein samples were resolved by SDS-PAGE gel electrophoresis, and they were transferred to a PVDF 0.45 μm membrane (Millipore) and blocked with 5% milk solution in Tris buffer solution (TBS). Membranes were incubated with the corresponding primary antibody (Table 4) diluted in 3% BSA solution for 16 h at 4ºC. After that, membranes were washed three times in TBS with 0,05% Tween-20 and were incubated with the corresponding secondary antibody. Blots were revealed using Pierce ECL Plus Western Blotting Substrate (Thermo Fisher) and bands were obtained using ImageQuant LAS 4000 machine (GE Healthcare Life Science). Finally, bands were quantified using ImageJ software and the values were normalized to GAPDH.

**Table 4:** List of antibodies used for protein detection

Antibody	Reference	Dilution	Description
**Anti-RhoE/Rnd3** **(Sigma-Aldrich)**	05-723	1:200	Mouse monoclonal primary antibody
**Anti-GAPDH**	sc-47,724	1:500	Mouse monoclonal primary antibody
**Anti-Mouse IgG-HRP (Fc)** **(Thermo Fisher)**	31,437	1:5000	Secondary antibody conjugated with HRP

### Transcriptomic analysis

To validate the impact of Rnd3 absence, bulk population cells obtained after puromycin selection were used for a transcriptomic analysis consisting of 3’ UTR RNA sequencing, as previously described [34]. Bioinformatics tool LIMMA [35] was used to identify the genes with significant differential expression between experimental conditions (sgRND3 #3 or sgRND3 #4 compared to control samples in RPMI8226 or JJN3 cell lines). Genes were selected as differentially expressed using a p-value cut off p < 0.05 only when the observed logFC was significant and coherent in the results of both cell lines. Clustering analyses and graphical representations were performed using R/Bioconductor [36] and clusterProfiler [37].

### Statistical analysis

Data were expressed as a mean ± SEM. The statistical analysis was performed using the GraphPad Prism (GraphPad software). Statistical comparison among groups was evaluated using one-way ANOVA and Tuckey post-hoc test. In all cases, p < 0.05 was considered statistically significant.

## Electronic supplementary material

Below is the link to the electronic supplementary material.

Supplementary Material 1

## Data Availability

The data that support the findings of this study are available on request from the corresponding author.

## Abbreviations

MM: Multiple myeloma
RNAi: RNA interference
CRISPRi: CRISPR interference
MGUS: Monoclonal gammopathy of undetermined significance
SMM: Smoldering multiple myeloma
EMM: Extramedullary multiple myeloma
PCL: Plasma cell leukemia
sgRNA: Single guide RNA
ORF: Open reading frame
HR: Homology repair
NHEJ: Nonhomologous end joining
dCas9: Dead Cas9
KRAB: Krüppel-associated box
HEK 293T: Human embryonic kidney 293T cells
FBS: Fetal bovine serum
TBS: Tris buffer solution
KD: Knock-down
