# Supplementary figures and images for: A CRISPR interference strategy for gene expression silencing in multiple myeloma cell lines
Source: J Biol Eng. 2023 May 4;17:34. doi: 10.1186/s13036-023-00347-7 (PMC10161638; doi:10.1186/s13036-023-00347-7)

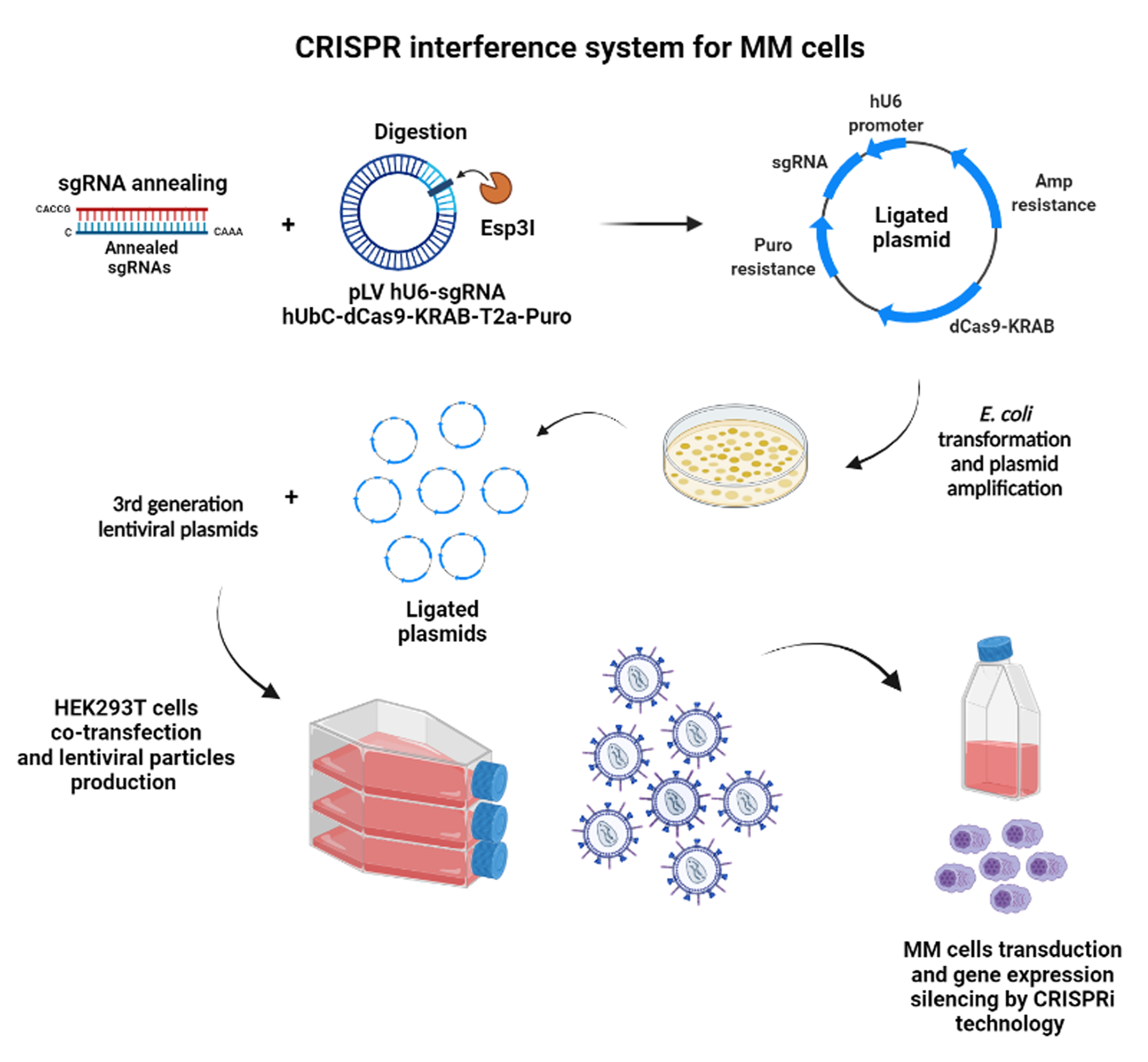

Supplement: Supplementary file 1 — Supplementary Material 1 [file 13036_2023_347_MOESM1_ESM.tif]
